# High proportion of thiamine deficiency in referred cancer patients with delirium: a retrospective descriptive study

**DOI:** 10.1038/s41430-021-00859-9

**Published:** 2021-01-29

**Authors:** Hideki Onishi, Izumi Sato, Nozomu Uchida, Takao Takahashi, Daisuke Furuya, Yasuhiro Ebihara, Akira Yoshioka, Hiroshi Ito, Mayumi Ishida

**Affiliations:** 1 Department of Psycho-oncology, Saitama Medical University International Medical Center, 1397-1 Yamane, Hidaka City, Saitama, 350-1298 Japan; 2 Department of Pharmacoepidemiology, Graduate School of Medicine and Public Health, Kyoto University, Kyoto, Japan; 3 Department of General Medicine, Ogano Town Central Hospital, Ogano, Japan; 4 Department of Supportive Medicine, Saitama Medical University International Medical Center, Hidaka, Japan; 5 Department of General Medicine, Saitama Medical University International Medical Center, Hidaka, Japan; 6 Department of Laboratory Medicine, Saitama Medical University International Medical Center, Hidaka, Japan; 7 Department of Clinical Oncology, Mitsubishi Kyoto Hospital, Kyoto, Japan; 8 Ito Internal Medicine and Pediatric Clinic, Fukuoka, Japan; 9 Department of Psycho-oncology, Saitama Medical University International Medical Center, Hidaka, Japan

**Keywords:** Endocrine system and metabolic diseases, Metabolic disorders

## Abstract

**Background/Objectives:**

Recent studies have revealed thiamine deficiency (TD) as a cause of delirium in cancer patients. However, the extent to which Wernicke encephalopathy is present and in what patients is not well understood.

**Subjects/Methods:**

In this retrospective descriptive study, we investigated referred cancer patients who were diagnosed with delirium by a psycho-oncologist to clarify the proportion of TD, the therapeutic effect of thiamine administration, and the factors involved in its onset.

**Results:**

Among 71 patients diagnosed with delirium by a psycho-oncologist, TD was found in 45% of the patients. Intravenous administration of thiamine led to a recovery in about 60% of these patients. We explored the factors associated with TD using a multivariable regression model with a Markov chain Monte Carlo imputation procedure. We found an association between TD and chemotherapy (adjusted odds ratio, 1.98 [95% confidence interval, 1.04–3.77]); however, there were no significant associations between TD and the other factors we considered.

**Conclusions:**

TD is not particularly rare in delirium patients undergoing psychiatric consultation. The delirium was resolved in more than half of these patients by intravenous administration of thiamine. Oncologists should consider TD as a cause of delirium in cancer patients. Further prospective study is needed to clarify the relationship between TD and delirium in cancer patients.

## Introduction

Delirium is one of the major problems arising during the course of cancer treatment. As this condition is common in patients with advanced cancer and is associated with increased complications and mortality, an examination of its causes in its early stages as well as therapeutic interventions is required [1]. However, delirium is often unrecognized or misdiagnosed by physicians [2]. According to a survey of cancer patients, delirium was missed in 50–60% of cases [3, 4]. In addition, recent studies have reported thiamine deficiency (TD) as a cause of delirium in cancer patients [5–9].

Thiamine is a water soluble vitamin and, in its biologically active form of thiamine pyrophosphate, is an essential coenzyme for oxidative metabolism [10]. This vitamin cannot be synthesized in vivo and depends on external intake. The recommended daily allowance of thiamine is 1.2 mg/day for men and 1.1 mg/day for women [11, 12]. Furthermore, the population reference intake is 0.4 mg/1000 kcal [13]. However, since stores in the body are exhausted within 18 days [14], deficiency can occur when a patient experiences a loss of appetite lasting a few weeks.

Various issues have been highlighted regarding nutritional intake in cancer patients. A study comparing dietary intake in patients undergoing chemotherapy for gastric cancer before and at 6 months after the start of chemotherapy showed a significant decrease in vitamin B1, B6, and B12 intake, as well as significant decreases in the consumption of beef, low-fat milk, and raw vegetables [15]. A survey of patients receiving chemotherapy for breast cancer found problems with dietary intake in 80% of patients [16]. Patients with neuroendocrine tumors were found to have lower intake of vegetables, fruits, wine, seafood, and nuts, and increased intake of meat, butter, meat, margarine, soda, etc., compared to controls. In addition, patients with aggressive disease showed lower adherence to a Mediterranean diet.

A typical neuropsychiatric disorder associated with TD is Wernicke encephalopathy (WE), which is recognized by a classic triad of symptoms: mental status changes, ataxia, and ocular symptoms. This disease was previously seen as a common illness associated with alcoholism, but it can develop in association with any disease resulting in a loss of appetite [17]. Indeed, it is not a rare disease and, in a prospective necropsy study, the prevalence of the Wernicke-Korsakoff syndrome in Sydney, Australia was shown to be 2.1% of adults over the age of 15 years [18]. Recent research has shown that the disease is also common in cancer patients. Onset varies from initial chemotherapy treatment, to post-surgery, to treatment with anticancer drugs for recurrent cancer, to end-stage cancer [5–9, 19–21].

The treatment for TD, including WE, involves the intravenous administration of thiamine [22] and, if it is detected early, patients can recover without any sequelae. However, failure to treat the condition early due to it being unrecognized or misdiagnosed can result in Korsakoff syndrome, leading to severe and irreversible brain damage [10]. The mortality rate for Korsakoff syndrome is as high as 17% [23]. However, WE is often overlooked or is misdiagnosed as another mental illness [9, 10, 17, 24] as the individual symptoms are not disease-specific, and only 16% cases present with all three classic symptoms of WE, with those without any of the three symptoms representing nearly 19% of cases [25]. Therefore, in cancer patients with delirium, early detection and treatment of TD is an important issue for preventing severe brain damage, such as that associated with Korsakoff syndrome, and maintaining the patient quality of life. However, the extent to which and in what patients WE is present is not well understood.

Therefore, we undertook this retrospective descriptive study of referred cancer patients who developed delirium, focusing on the proportion of those with TD and their characteristics.

## Methods

### Participants and procedures

#### Study design and setting

We conducted a retrospective descriptive study using electronic medical records (EMRs) for patients with cancer referred to the Department of Psycho-oncology, Saitama Medical University International Medical Center, and diagnosed with delirium between October 1st, 2014 and May 31th, 2018. The Cancer Center is in a university hospital with 400 beds that treats about 4800 new patients each year and is the highest volume cancer center in any Japanese university hospital. Our department handles about 5% of the patients who visit the Cancer Center.

### Subjects

#### Inclusion criteria

We included patients with cancer aged 18 years or older who were diagnosed with delirium on consultation with a psycho-oncologist who was a psychiatrist (psycho-oncologist is a collective term for medical professionals and researchers specializing in the clinical treatment and research of cancer) with extensive experience with cancer patients. Delirium screening was performed for all (*n* = 714) patients referred, the reason being that the doctor in charge often misdiagnoses delirium [26].

The criteria set in the Diagnostic and Statistical Manual of Mental Disorders 5th edition (DSM-V) were used to diagnose delirium [27]. These diagnostic criteria, published by the American Psychiatric Association, are the most commonly used diagnostic criteria around the world, and help resolve problems with the reliability of diagnosis among psychiatrists. These diagnostic criteria are also used as part of routine clinical care in psychiatric settings around the world. When a diagnosis of delirium could not be confirmed, we conducted multiple examinations and follow-ups, and also conferred with psychological specialists who are also psycho-oncologists (MIs) experienced in treating cancer patients, before making a final diagnosis. All cases were finally determined to be delirium through consultation with psychological specialists (MIs).

### Exclusion criteria

We excluded patients receiving oral or intravenous vitamin administration, those who met the DSM-V criteria for alcohol use disorders, those whose health status has deteriorated, those for whom blood collection was impossible, or those for whom, in the researcher’s judgment, blood collection was inappropriate.

### Data collection from electrical medical records

We collected data regarding each patient’s background (age, gender, height, weight, primary site of cancer, pre-existing disease, and Performance Status [PS]), history of cancer treatment [radiation/chemotherapy/surgery/hormone] within 2 months from the time of blood sampling for vitamin B1 measurement, and disappearance of the symptoms of delirium after vitamin B1 treatment from EMRs, and results of laboratory examinations (vitamin B1 level, albumin level, and lactate dehydrogenase [LDH] level), which were ordered as part of routine clinical care based on the judgment of the treating physicians at the time of referral or within a few days before consultation. Performance status (PS), as defined by the Eastern Cooperative Oncology Group criteria provides an objective index of a patient’s physical functioning on a scale of 0 to 4, with a higher score indicating greater physical condition deterioration (PS = 0 indicates no symptoms, PS = 4 indicates completely bedridden) [28]. Presence or absence of delirium was judged comprehensively from the description of delirium in the medical records and examination by the psycho-oncologist. Serum thiamine concentration was measured using high-performance liquid chromatography at an outside laboratory (SRL, Inc. Tokyo). This method is precise and rapid, and is less susceptible to factors that alter enzyme activity, while providing a more sensitive assay for screening TD in a clinical setting and for research purposes [29].

### Statistical analysis

The sample size was determined to provide adequate power for the assessment of the outcomes of TD induced by chemotherapy. We expected a difference in the proportion of patients (0.35) with TD induced by chemotherapy. We calculated that a sample size of 60 patients (30 patients in each group) would provide 80% power to detect an odds ratio of 1.75, using a two-sided type I error rate of 0.05.

We calculated descriptive statistics to summarize the patients’ background, history of cancer treatment and results of laboratory examinations. We set an albumin level under 3.9 g/dl as representing low albumin, LDH under 212 U/L as low LDH, serum thiamine concentration under <24 ng/ml as thiamine deficient, and BMI under 90% of average as low BMI. We then performed univariable and multivariable logistic regression analyses to investigate the factors associated with TD among the following factors: age, sex, BMI, LDH, albumin, gastroenterological cancer, performance status, cancer treatment (radiation/chemotherapy/surgery/hormone), and comorbidities (diabetes mellitus, high-blood pressure, and cardiac disorder). We imputed BMI, LDH, albumin, and PS when we estimated the adjusted odd ratios using the Markov chain Monte Carlo method (Yuan [Ed], *Multiple imputation for missing data: Concepts and new development. Proceedings of the Twenty-Fifth Annual SAS Users Group International Conference*; 2000). All statistical operations were performed with SAS statistical software version 9.4 for Windows (SAS Institute Inc., Cary, NC, USA). All p values were two-sided, and the α level was set at 0.05.

The ethical and scientific validity of this study was approved by the institutional review board of Saitama Medical University International Medical Center (18–080).

## Results

During the study period, the number of consultation requests for cancer patients was 714, of which 143 were diagnosed with delirium.

**Fig. 1:**
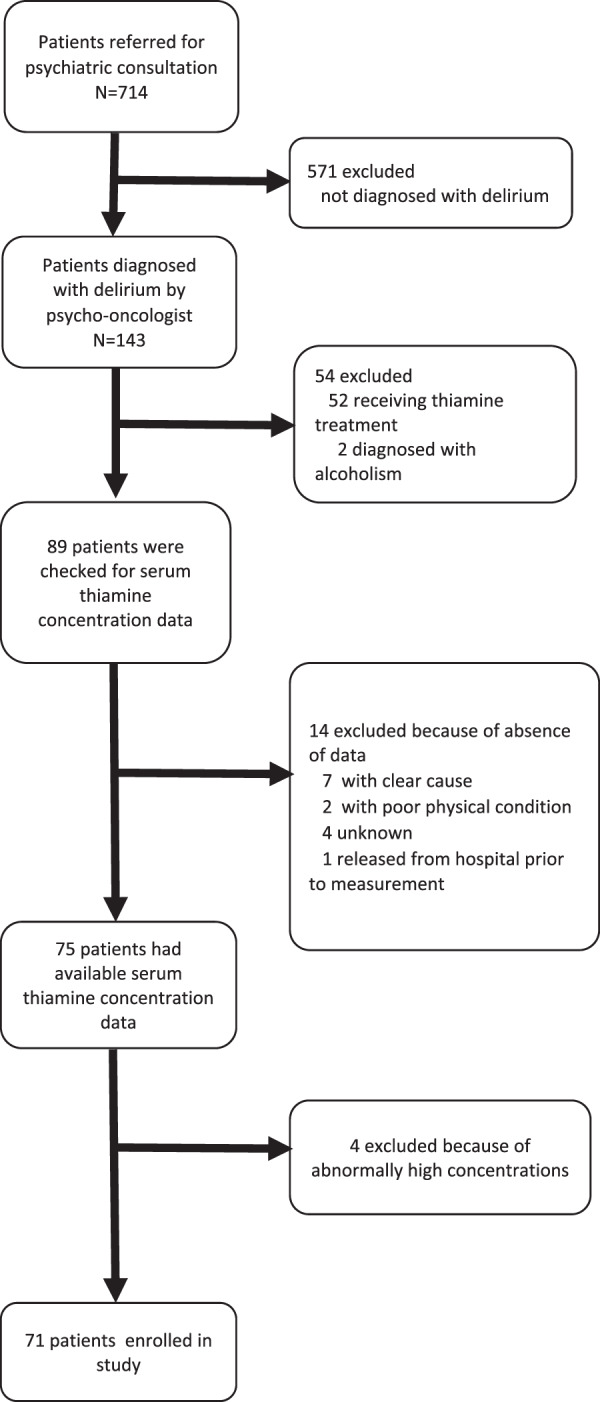
Patient selection.

Of these 143 patients diagnosed with delirium, 71 patients satisfied the eligibility criteria (Fig. 1). We excluded 72 patients for the following reasons; 52 were treated with thiamine, two were diagnosed with alcoholism, 14 had no blood test results, and four with thiamine levels regarded as outliers.

The study cohort comprised 66% male patients with a mean age (SD) of 69.6 (±10.4) years (Table 1). Of the 71 patients, 45.1% (*n* = 32) had TD. The mean age (SD) in patients with TD and patients with normal thiamine levels was 68.9 (10.8) and 70.3 (9.8), respectively. Also the mean albumin level (SD) was 3.0 (0.6) g/dL, and 3.3 (0.7) g/dL, mean LDH was 478.0(447.0) U/L and 396.0 (404.2) U/L, and mean BMI (SD) was 21.1 (3.3) and 20.6 (2.7), respectively. Patients with TD were more likely to have a BMI under 90% of average, to have gastroenterological cancer and comorbidities, and to have received cancer treatment within 2 months.

**Table 1:** Patients’ characteristics.

Variable	Total *n* = 71		Thiamine deficiency *n* = 32	Normal Thiamine level *n* = 39
	n (%)		n (%)	n (%)
Male	44(62.0)		19(59.4)	25(64.1)
Age(years)				
Mean (SD)		69.6(10.4)	68.9(10.8)	70.3(9.8)
min-max		35–86	35–86	46–86
Albumin (n = 65)				
<4.5 g/dL		57(87.7)	28(90.3)	29(78.4)
Mean (SD)		3.1(0.7)	3.0(0.6)	3.3(0.7)
min-max		1.9–4.7	1.9–4.6	1.9–4.7
LDH (n = 65)				
<212 U/L		17(26.2)	7 (22.6)	10(29.4)
Mean (SD)		422.5(432.2)	478.0(477.0)	396.0(404.2)
min-max		137–2,125	137–2,125	138–2,098
Body Mass Inde(BMI) (*n* = 62)				
<90% average		34(54.8)	18(60.0)	16(50.0)
Mean (SD)		20.7(3.1)	21.1(3.3)	20.6(2.7)
min-max		12–30	12–30	15–29
Gastroenterological cancer		14(19.7)	8(25.0)	6(15.4)
Performance status (*n* = 67)				
0		3(4.5)	0(0.0)	3(7.9)
1		6(9.0)	2(6.9)	4(10.5)
2		18(26.9)	9(31.0)	9(23.7)
3		29(43.3)	13(44.8)	16(42.1)
4		11(16.4)	5(17.2)	6(15.8)
Cancer treatment				
Received treatment within two months		52(73.2)	27(84.4)	25(64.1)
Hormone therapy		3(4.2)	2(6.3)	1(2.6)
Radiation therapy		14(19.7)	6(18.8)	8(20.5)
Chemotherapy		31(43.7)	20(62.5)	11(28.2)
Surgery		14(19.7)	6(18.8)	8(20.5)
Comorbidity				
Diabetic mellitus		8(11.3)	4(12.5)	4(10.3)
High-blood pressure		16(22.5)	9(21.9)	7(17.9)
Cardiac disorder		5(7.0)	3(6.3)	2(5.1)

BMI (Body Mass Index) = kg/m^2^, where kg is a person’s weight in kilograms and m^2^ is their height in meters squared.

Intravenous administration of thiamine was performed for all TD patients (*n* = 32), with a recovery observed in 59.3% of the patients.

We found only an association between TD and chemotherapy (adjusted odds ratio, 1.98 [95% confidence interval, 1.04–3.77]); however, there were no significant associations between TD and any of the other factors we considered (Table 2).

**Table 2:** Odds ratio for thiamine deficiency among patients with delirium.

variable	OR	95%CI	*P* value	aOR	95%CI	*P* value
Age	0.99	(0.94–1.04)	0.62	1.00	(0.93–1.06)	0.9
Sex (ref = female)	0.57	(0.21–1.54)	0.27	0.67	(0.36–1.25)	0.21
Albumin (ref = <4.5 g/dL)	0.39	(0.08–1.50)	0.19	0.56	(0.24–1.31)	0.18
BMI (ref = <90% average)	1.17	(0.42–3.24)	0.77	1.29	(0.69–2.42)	0.42
LDH (ref = >211 U/L)	1.00	(1.00–1.00)	0.33	1.33	(0.65–2.73)	0.44
Gastroenterological cancer	1.83	(0.57–6.24)	0.31	1.53	(0.68–3.45)	0.31
Performance status(ref = 0)	1.29	(0.79–2.17)	0.32	1.32	(0.73–2.37)	0.36
Hormone therapy	2.53	(0.23–56.01)	0.46	2.32	(0.51–10.63)	0.28
Radiation therapy	0.89	(0.26–2.90)	0.85	0.9	(0.43–1.89)	0.79
Chemotherapy	4.24	(1.60–11.92)	0.00	1.98	(1.04–3.77)	0.04
Surgery	0.89	(0.26–2.90)	0.85	0.85	(0.34–2.12)	0.73
Diabetic mellitus	1.25	(0.27–5.72)	0.77	1.14	(0.37–3.55)	0.82
High-blood pressure	1.79	(0.58–5.68)	0.31	1.35	(0.65–2.76)	0.42
Cardiac disorder	1.91	(0.30–15.26)	0.49	1.62	(0.47–5.58)	0.45

*OR* odd ratio, *aOR* adjusted odd ratio, *BMI* body mass index, *LDH* lactate dehydrogenase.

## Discussion

In this retrospective descriptive study, we clarified the percentage of cancer patients with delirium who experienced TD as well as the patients’ background characteristics. We found TD in about 45% of cancer patients diagnosed with delirium who were referred for a psychiatric examination and had not received thiamine. The TD in these patients was associated with receiving chemotherapy two months prior to blood sampling. In addition, the symptoms of delirium were improved in half of these patients by the intravenous administration of thiamine. These results indicate that TD is not uncommon in referred cancer patients diagnosed with delirium.

This study also clarified several problems.

Thiamine deficiency was found in 45% of the delirium patients enrolled. A study of referred patients examined at cancer center hospital in the United States found that 55% had TD [30]. Thus, our findings supported those of the US study, and appear to indicate that TD may not be recognized by oncologists as the cause of delirium.

Furthermore, we found an association between TD and chemotherapy. The reason why chemotherapy is related to TD is related to the patients’ medical condition and treatment. Based on the previous literature and our clinical observations [10, 14, 31–33], we hypothesized that an appetite loss lasting more than 2 weeks, a side effect of chemotherapy, which also involves the over utilization and destruction of thiamine, leads to TD. However, the other factors we considered were not associated with TD. As this study was a retrospective descriptive study, it cannot be ruled out that the failure to collect blood in all cases may have had an effect. It seems that the number of patients diagnosed with TD in this study was insufficient for meaningful statistical analysis. It will be necessary to perform a prospective study in the future.

In this study, patients with delirium were considered, but some patients with TD do not exhibit delirium [24, 25, 30, 34, 35]; therefore, even more patients with TD may be identified in future detailed investigations.

The limitations of this study include that fact that it was a single-center study targeting patients with delirium who consulted a psychiatrist. This study has referral bias as it was focused on cases referred for psychiatric consultation. Thus, the ability to draw generalizations from this study is also limited. Another limitation in this study was that we were able to identify patients with alcohol dependence, but were unable to clarify their alcohol intake in detail. Furthermore, serum thiamine concentration was not measured in all patients who received consultations. It might be that only patients displaying relatively severe symptoms received blood tests. In addition, there may be a lack of measurement of confounding factors related to TD, as we did not consider the history of medication use and all comorbidities. Further investigation is still needed using a database, including variables such as medication use and comorbidities, for a large population from multiple centers.

In conclusion, it was found that TD is not particularly rare in delirium patients undergoing psychiatric consultation. Further research is expected to reveal the actual situation regarding TD in cancer patients, enabling the prevention of WE and Korsakoff syndrome, and contributing to improved quality of life for cancer patients.
